# The impact of orthodontic-surgical treatment on female patients’ temporomandibular symptoms, psychological distress, and quality of life

**DOI:** 10.1093/ejo/cjaf062

**Published:** 2025-07-31

**Authors:** Elli Suomela, Outi Alanko, Martti T Tuomisto, Erkki Svedström, Timo Peltomäki, Auli Suominen, Tero Soukka, Anna-Liisa Svedström-Oristo

**Affiliations:** Pediatric Dentistry and Orthodontics, Institute of Dentistry, University of Turku, FI-20014,Turku, Finland; Pediatric Dentistry and Orthodontics, Institute of Dentistry, University of Turku, FI-20014,Turku, Finland; Faculty of Social Sciences (Psychology), Tampere University, FI-33014,Tampere, Finland; Diagnostic Radiology, Turku University Hospital, University of Turku, Kiinamyllynkatu 4-8, FI-20520, Turku, Finland; Faculty of Medicine and Health Technology, Tampere University, FI-33014, Tampere, Finland; Department of Community Dentistry, Institute of Dentistry, University of Turku, FI-20014, Turku, Finland; Department of Oral Diseases, Turku University Hospital, PO Box 52, FI-20521, Turku, Finland; Pediatric Dentistry and Orthodontics, Institute of Dentistry, University of Turku, FI-20014,Turku, Finland

**Keywords:** MRI, orthognathic surgery, psychological distress, quality of life, satisfaction

## Abstract

**Objective:**

To analyze changes in patients’ temporomandibular dysfunction (TMD) symptoms during orthodontic-surgical treatment and to investigate associations between symptoms, findings in temporomandibular joints (TMJs), satisfaction with occlusal function, psychological distress, and orthognathic quality of life (OQoL).

**Subjects and Methods:**

Thirty-six consecutive female patients started orthodontic-surgical treatment, and 28 voluntary controls participated. Patients and controls filled in a semi-structured diary (regarding satisfaction and TMD symptoms) and the Orthognathic Quality of Life Questionnaire (OQLQ); patients also filled in the Symptom Checklist-90 (SCL-90). Patients’ data were analyzed before treatment (T1), after preoperative orthodontics (T3), and one year after jaw surgery (T5). Controls’ data were collected at respective time points (CT1, CT2, CT3). Magnetic Resonance Imaging (MRI) studies were performed on patients’ and controls’ TMJs at T1/CT1.

**Results:**

Before surgery, patients reported more TMD symptoms than controls. The most frequent symptoms were head/neck pain, stiffness/fatigue of the jaws, and pain in jaw joints. The most common finding in TMJ-MRI was anterior disc displacement with or without reduction. One year after surgery, improvement was seen in patients’ satisfaction and OQLQ scores. Patients’ number of symptoms correlated negatively with satisfaction and positively with OQLQ scores. No significant correlations were found between the severity of TMJ-MRI findings and satisfaction, number of symptoms, OQLQ function, or sum score.

**Conclusions:**

Although TMJ-MRI findings are common among orthodontic-surgical patients, they are not always reflected in subjective symptoms, nor in self-perceived treatment outcome. Individual characteristics, not detectable using objective measures, constitute an important aspect and should therefore reserve more emphasis.

## Introduction

Combined orthodontic-surgical treatment focuses on the correction of severe skeletal discrepancies that cannot be treated by orthodontic growth modification or camouflage only after growth has ceased. In addition to creating a more harmonious facial appearance, orthodontic-surgical treatment aims to improve patients’ dentofacial function and health-related quality of life. Traditionally, treatment consists of three phases: preoperative orthodontics, jaw surgery, and postoperative orthodontics [1]. According to recent studies, the approximate duration of active treatment is 30 months [2, 3].

The most common reasons for seeking orthodontic-surgical treatment are aesthetic and functional, although there is wide variation; 11%–95% of patients have reported aesthetic reasons [4–8] and 23%–85% functional reasons [4, 6–9]. In addition, the effects of malocclusion on awareness of one’s appearance and self-esteem have been reported as reasons for seeking treatment [10].

The most common functional reasons include biting, chewing, and eating problems [6], while other, less often reported reasons involve problems in speech or pronunciation and issues in the nose or throat [4, 6]. In the study by Pahkala et al. [8], the most frequently mentioned reasons were regular headaches, facial pain, and problems in temporomandibular joints (TMJs). Furthermore, orthodontic-surgical treatment has also become an integral part of treatment for obstructive sleep apnea (OSA) [11]. According to the meta-analysis by Al-Moraissi et al. [12], patients with dentofacial deformity have a higher prevalence of temporomandibular dysfunction (TMD) symptoms than healthy controls. Also, in earlier studies, symptoms like headache, masticatory muscle tenderness, pain, and clicking in TMJs have been reported by preoperative orthognathic patients [13, 14].

Although the impact of orthodontic-surgical treatment on patients’ TMD symptoms is controversial [12, 13, 15–18], most patients report satisfaction with the treatment outcome [8, 9, 11, 19]. Positive effects on patients’ maximum bite force, facial appearance, and self-esteem have been reported in several studies [9, 10, 18–20].

This cross-sectional study had two aims: (1) to analyze changes in patients’ self-reported TMD symptoms during orthodontic-surgical treatment and (2) to investigate associations between findings in TMJs, TMD symptoms, satisfaction with occlusal function, psychological distress, and orthognathic quality of life.

## Subjects and methods

### Patients

The study subjects comprised 36 consecutive female patients (mean age 33.7 years, range 18.3–54.9 years) starting orthodontic-surgical treatment at the Department of Oral and Maxillofacial Diseases of Turku University Hospital or the Oral and Maxillofacial Unit of Tampere University Hospital. Exclusion criteria comprised cleft lip and/or palate, any syndrome affecting the craniofacial complex, and insufficient Finnish language skills.

The patients were requested to fill in a semi-structured diary created by the authors. It contained questions about daily activities, satisfaction with own occlusal function, and, a semi-structured follow-up question about causes for possible dissatisfaction [21]. The applied TMD-related statements/questions from the Research Diagnostic Criteria for Temporomandibular Disorders (RDC/TMD) History Questionnaire [22] and the Diagnostic Criteria for Temporomandibular Disorders (DC/TMD) Symptom Questionnaire [23] were slightly modified to help the respondents focus on their current experience. Satisfaction with own occlusal function was assessed using a scale from 1 to 7 (1 = highly dissatisfied, 7 = highly satisfied). At each time point, the diary was to be filled in four times in one day: after waking up in the morning (at 8 am), after lunch (at noon), after work (at 4 pm), and before going to sleep (at 8 pm). In addition to the diary, patients filled in two questionnaires: (1) Symptom Checklist-90 (SCL-90) [24] and (2) Orthognathic Quality of Life Questionnaire (OQLQ) [25]. The diary and the questionnaires were filled in as soon as possible after the appointment.

The OQLQ includes 22 questions categorized into four subcategories: oral function, facial aesthetics, awareness of facial aesthetics, and social aspects of dentofacial deformity. Higher scores indicate poorer orthognathic quality of life (OQoL) [25]. The SCL-90 [24] is a self-report questionnaire of psychiatric symptoms. It consists of 90 items, which form nine subscales: somatization, compulsivity, interpersonal sensitivity, depression, hostility, anxiety, phobic anxiety, paranoid ideation, and psychoticism. Higher scores indicate higher symptom intensity. The Global Severity Index (GSI, ‘psychological distress’) reflects the mean score of all the SCL-90 items. The SCL-90 and OQLQ are used widely, and their validity and reliability have been tested [25, 26]. In addition to the questionnaires, 34 pairs of acceptable pretreatment study models were available for analysis. One calibrated orthodontist analyzed the models using the Index of Complexity, Outcome, and Need (ICON) [27]. The reliability was high (Cronbach’s α 0.829, Spearman’s rho 0.700, p = 0.016).

### Controls

The control group was set up to find out what changes emerge in predominantly healthy adults’ TMD-related symptoms as a function of time. The follow-up time was chosen to respond to the follow-up of orthognathic-surgical treatment. The control group comprised 28 voluntary university students, all females (mean age 24.9 years, range 19.5–49.0 years) recruited from the Turku unit of the Finnish Student Health Service. Before the study, they all attended a dental and oral examination. None of the controls reported TMJ symptoms or needed orthodontic or orthognathic treatment. The controls filled in the semi-structured diary and the OQLQ according to the exact instructions to the patients.

### Study protocol

For patients, there were seven data collection points: before treatment (T0), after the first appointment (T1), 6–8 weeks after the placement of orthodontic appliances (T2), after preoperative orthodontics (T3), six weeks after surgery (T4), one year after surgery (T5), and after completion of postoperative orthodontics, including retention (T6, 20–57 months after surgery). This article analyses patients’ and controls’ diaries, OQLQ function and sum scores, and patients’ responses to SCL-90 at time points T0–T1, T3, and T5. The longitudinal results of the prospective study have been reported previously [28, 29].

Patients’ first questionnaires were mailed together with information about their first appointment (T0); the first diary with instructions was given after this appointment (T1). Later, the questionnaires and diaries were given after each appointment in accordance with the study protocol.

Controls’ data were collected at three time points: after attending the dental and oral examination (CT1), two years later (CT2, corresponding to patients’ T3), and four years after the first data collection point (CT3, corresponding to patients’ T5). All questionnaires and diaries were sent by mail.

At T1/CT1, all patients and controls were provided with a 3.0 Tesla TMJ-MRI study. Standard non-contrast TMJ protocols with 2D and 3D sequences without surface coil were used. Both the mouth-open and mouth-closed sequences were obtained. The MRI findings (the disc form, signal, disc dislocation with or without reduction, as well as the condyle position, shape and bony changes, the lateral pterygoid muscle and the joint fluid) were evaluated by one experienced radiologist. The pilot study measuring the intra-observer agreement showed that it was good or excellent except for the classification of the disc signal. The findings were classified according to the system by Hegab et al. [30]. In this system, the disc position in MRI is classified into one of three stages: (1) no disc displacement, (2) anterior disc displacement, and (3) posterior disc displacement. These stages are further divided into five substages 0–4, describing all the pathological changes in the joint. This study reports the results for patients and controls and separately for the joints. A description of the MRI findings included in stages 0–4 is presented in Table 1.

**Table 1.** Classification of the temporomandibular joints’ MRI findings according to the system by Hegab et al. [30].

	STAGES 0–1			STAGE 2			STAGE 3			STAGE 4
FINDINGS IN MRI	*Stage 0*	*Sub-stage 1A*	*Sub-stage 1B*	*Sub-stage 2A*	*Sub-stage 2B*	*Sub-stage 2C*	*Sub-stage 3A*	*Sub-stage 3B*	*Sub-stage 3C*	*Sub-stage 4*
Normal condyle-disc relationship ^1^ADDwr ^2^ADDwnr ^3^PDDwr ^4^PDDwnr	x	x	x	x	x	x	x	x	x	x x
Normal lateral pterygoid muscle Fatty degeneration Hypertrophy or fatty degeneration or atrophy	x	x x	x x	x x	x x	x x x x	x x	x x x x	x x x x	
No joint effusion Joint effusion	x	x	x x	x x	x x	x x	x x	x x	x x	
Bone degenerative process Severe bone degenerative process			x		x			x	x	x
Disc degeneration			x		x			x	x	x
Stuck disc			x		x			x		
Pseudo disc formation									x	
Disc perforation or pseudo disc			x		x				x	x
Condylar hypertranslation						x				

^1^Anterior Disc Displacement with reduction.

^2^Anterior Disc Displacement without reduction.

^3^Posterior Disc Displacement with reduction.

^4^Posterior Disc Displacement without reduction.

The number of patients at T1 was 36, at T3 19, and at T5 13. The control group comprised 28 university students at CT1, 26 at CT2, and 22 at CT3.

### Statistical methods

The Mann-Whitney U-test was used to compare patients’ and controls’ satisfaction with occlusal function, OQLQ function and sum scores, and number of symptoms. Patients’ GSI values were compared to the national value [26]. Proportions of symptomatic and non-symptomatic patients and controls were compared with the Likelihood–Ratio Test (LRT). Correlations between satisfaction with occlusal function, number of symptoms, GSI, OQLQ function and sum scores, and MRI findings were calculated with Spearman’s correlation coefficient (rho). All p-values below 0.05 were considered statistically significant.

### Power calculation

The current study compared two independent groups in a cross-sectional study design. A post hoc power analysis (Student t-test, two-sided, α = 0.05) was conducted for this cross-sectional study based on the means and standard deviations of the OQLQ function scores. The mean and standard deviation were derived separately for patient and control groups at each measurement point, using the corresponding group sizes at each time point.

With a significance level of α = 0.05, the power at T0 and T3 was 99%, while at T5, it was 5%.

The calculation was repeated using the mean and SD of Satisfaction with occlusal function, maintaining α = 0.05. This resulted a power 100% at T0 and T3, and 4% at T5.

### Ethical approval

The Ethics Review Committee of the Hospital District of Southwest Finland and the Joint Municipal Authority of the Pirkanmaa Hospital District approved the study protocol (Dno. ETMK 80/180/2009 9/2009 §361 and 3/2010 §67). All the participants signed informed consent before the study, and participation was voluntary.

## Results

### Background

The main reasons for seeking orthodontic-surgical treatment were headache (41%) and problems related to TMD (30%), followed by traumatic deep bite (22%), and muscular pain (22%). One in six patients mentioned problems with eating, aesthetics and sleep apnea, and snoring. Of all patients (n = 36), 69% were diagnosed with mandibular retrognathia. Of them, 20% had problems with deep bite, 4% with open bite, 28% with overjet, and 28% with both overjet and overbite. Maxillary retrognathia was diagnosed with or without mandibular prognathia in 14% of the patients. Other diagnoses included dental problems (11%) and skeletal and dental asymmetries (6%).

In the analysis of study models (n = 34), the mean ICON score was 61 (median 63, range 13–94). The ICON score in 82% of models exceeded 43, indicating orthodontic treatment need. The complexity of orthodontic treatment was easy or mild in 32% (scores ≤ 50), moderate in 21% (scores 51–60) and difficult or very difficult (scores ≥ 64) in 47% of patients.

### Satisfaction with occlusal function

Before treatment (T1) and after preoperative orthodontics (T3), patients’ satisfaction with own occlusal function was lower than controls’, but one year after surgery (T5), no difference was found (Table 2). At T1/CT1 and T3/CT2, the difference between patients’ and controls’ satisfaction was statistically significant (p < 0.001 each). At T5, none of the patients reported dissatisfaction with occlusal function.

**Table 2.** Satisfaction with occlusion, OQLQ function score, and the number of TMD symptoms among patients and controls, and GSI among patients in the morning diary entries. Descriptive statistics and Mann-Whitney U-test before treatment (T0-T1/CT1), after presurgical orthodontics (T3/CT2), and one year after jaw surgery (T5/CT3).

	Measure	Group	n	Mean	SD	Median	Mann-Whitney U-test	P-value
T0-T1/CT1	*Satisfaction with occlusion*	Patient	36	3.39	1.76	3.00	U = 226.0	< 0.001
		Control	28	5.32	1.59	6.00		
	*OQLQ* *function*	Patient	33	11.85	4.64	12.00	U = 62.5	< 0.001
		Control	28	3.54	3.68	3.00		
	*Number of symptoms*	Patient	36	1.83	2.20	1.50	U = 324.5	0.006
		Control	28	0.46	0.96	0.00		
	*GSI*	Patient	36	0.62	0.51	0.44		
T3/CT2	*Satisfaction with occlusion*	Patient	19	3.37	1.38	3.00	U = 63.00	< 0.001
		Control	24	5.50	1.29	6.00		
	*OQLQ* *function*	Patient	19	13.79	4.48	14.00	U = 24.5	< 0.001
		Control	24	3.25	4.02	1.50		
	*Number of symptoms*	Patient	19	1.74	2.47	0.00	U = 140.5	0.007
		Control	24	0.21	0.59	0.00		
	*GSI*	Patient	19	0.67	0.66	0.42		
T5/CT3	*Satisfaction with occlusion*	Patient	13	5.69	1.25	6.00	U = 138.0	0.880
		Control	22	5.55	1.41	6.00		
	*OQLQ* *function*	Patient	13	3.92	3.40	3.00	U = 130.0	0.674
		Control	22	4.18	4.77	2.50		
	*Number of symptoms*	Patient	13	0.15	0.38	0.00	U = 135.0	0.801
		Control	22	0.50	1.10	0.00		
	*GSI*	Patient	13	0.30	0.26	0.22		

### Symptoms

At T1, 24 (66.7%) patients and 11 (39.3%) controls reported symptoms. The mean number of self-reported symptoms among patients was 4.29 (median 2, range 0–21) at T1, 5.16 (median 2, range 0–28) at T3, and 0.77 (median 0, range 0–6) at T5. Among controls, the respective values were 1.21 (median 0, range 0–9) at CT1, 1.00 (median 0, range 0–6) at CT2, and 1.73 (median 0, range 0–8) at CT3. At T5/CT3, two patients (15.4%) and eight controls (36.4%) reported symptoms.

The patients reported most symptoms in the morning. Their most frequent symptoms at T1 and T3 were head and/or neck pain, stiffness and/or fatigue of the jaws, chewing difficulties, and pain in the jaw joint. Among controls, the most frequently reported symptoms were jaw stiffness and/or fatigue and clicking of the jaw joints. After preoperative orthodontics at T3/CT2, in the Morning, Day 1, and Day 2 diary entries, a higher proportion of patients than controls reported symptoms. At T5/CT3, the proportion of patients with symptoms had decreased, and the difference between patients and controls was not statistically significant (Table 3). The detailed numbers of patients and controls reporting various symptoms are shown in Table 4.

**Table 3.** Percentages of symptomatic and non-symptomatic patients and controls according to the diary entries. Comparison of the groups with the Likelihood-Ratio Test (LRT). The numbers of patients at the three time points were n = 36 (before treatment, T1), n = 19 (after presurgical orthodontics, T3), and n = 13 (one year after jaw surgery, T5), and the numbers of controls n = 28 (CT1), n = 24 (CT2), and n = 22 (CT3), respectively.

DIARY			*PATIENTS* %	*CONTROLS* %	*LRT* (P)
Morning	T1/CT1	Symptomatic	52.8	25.0	5.17 (0.023)
		Non-symptomatic	47.2	75.0	
	T3/CT2	Symptomatic	47.4	12.5	6.55 (0.011)
		Non-symptomatic	52.6	87.5	
	T5/CT3	Symptomatic	15.4	18.2	0.046 (0.831)
		Non-symptomatic	84.6	81.8	
Day 1	T1/CT1	Symptomatic	36.1	10.7	5.82 (0.016)
		Non-symptomatic	63.9	89.3	
	T3/CT2	Symptomatic	42.1	8.3	7.01 (0.008)
		Non-symptomatic	7.9	91.7	
	T5/CT3	Symptomatic	15.4	18.2	0.046 (0.831)
		Non-symptomatic	84.6	81.8	
Day 2	T1/CT1	Symptomatic	27.8	14.3	1.65 (0.199)
		Non-symptomatic	72.2	85.7	
	T3/CT2	Symptomatic	36.8	8.3	5.34 (0.021)
		Non-symptomatic	63.2	91.7	
	T5/CT3	Symptomatic	15.4	18.2	0.046 (0.831)
		Non-symptomatic	84.6	81.8	
Evening	T1/CT1	Symptomatic	27.8	25.0	0.062 (0.803)
		Non-symptomatic	72.2	75.0	
	T3/CT2	Symptomatic	21.1	20.8	<0.001 (0.986)
		Non-symptomatic	78.9	79.2	
	T5/CT3	Symptomatic	15.4	22.7	0.284 (0.594)
		Non-symptomatic	84.6	77.3	

**Table 4.** Distribution of symptoms in the morning diary entries at time points T1/CT1, T3/CT2, and T5/CT3 among all patients and controls and according to the five stages 0–4 based on the severity of MRI findings (Hegab et al. [30]). None of the patients’ or controls’ joints were categorized into stage 4. The numbers of patients at the three time points were n = 36 (before treatment, T1), n = 19 (after presurgical orthodontics, T3), and n = 13 (one year after jaw surgery, T5), and the numbers of controls n = 28 (CT1), n = 24 (CT2), and n = 22 (CT3), respectively. The numbers of MRIs were n = 26 (patients) and n = 18 (controls).

		*ALL PATIENTS AND CONTROLS*		*STAGES 0–1* *uni- or bilaterally*		*STAGE 2* *uni- or bilaterally*		*STAGE 3* *uni- or bilaterally*	
SYMPTOM	Time point	Patients n (%)	Controls n (%)	Patients (n = 16) n (%)	Controls (n = 13) n (%)	Patients (n = 2) n (%)	Controls (n = 3) n (%)	Patients (n = 8) n (%)	Controls (n = 2) n (%)
Pain in the head and/or neck region	T1/CT1 T3/CT2 T5/CT3	16 (44) 7 (37) 1 (8)	1 (4) 2 (8) 2 (9)	7 (44) 4 (25) 0	0 0 0	1 (50) 1 (50) 0	0 0 0	2 (25) 1 (13) 1 (13)	1 (50) 0 1 (50)
Pain in the jaw joint	T1/CT1 T3/CT2 T5/CT3	9 (25) 5 (26) 0	1 (4) 0 2 (9)	3 (19) 2 (13) 0	0 0 1 (8)	1 (50) 1 (50) 0	0 0 0	1 (13) 1 (13) 0	0 0 1 (50)
Clicking or crepitation in the jaw joint	T1/CT1 T3/CT2 T5/CT3	7 (19) 3 (16) 0	3 (11) 0 0	2 (13) 1(6) 0	0 0 0	1 (50) 0 0	0 0 0	1 (13) 1 (13) 0	1 (50) 0 0
The jaw feels tired or stiff	T1/CT1 T3/CT2 T5/CT3	12 (33) 7 (37) 1 (8)	4 (14) 2 (8) 2 (9)	5 (31) 3 (19) 1 (6)	0 0 0	1 (50) 1 (50) 0	0 0 0	1 (13) 2 (25) 0	2 (100) 0 1 (50)
Difficulties opening	T1/CT1 T3/CT2 T5/CT3	4 (11) 2 (11) 0	1 (4) 0 2 (9)	1 (6) 0 0	0 0 1(8)	0 0 0	0 0 0	0 1 (13) 0	0 0 0
Teeth clenching or grinding	T1/CT1 T3/CT2 T5/CT3	6 (17) 3 (16) 0	2 (7) 1 (4) 1 (5)	2 (13) 0 0	0 0 0	1 (50) 1 (50) 0	0 0 0	0 1 (13) 0	0 0 0
Difficulties chewing	T1/CT1 T3/CT2 T5/CT3	10 (28) 5 (26) 0	0 0 1 (5)	3 (19) 2 (13) 0	0 0 1 (8)	0 0 0	0 0 0	2 (25) 2 (25) 0	0 0 0

### MRI findings

At T1/CT1, MRIs were obtained from 26 patients (72%) and 18 controls (64%). The most common findings were anterior disc dislocation with (ADDwr) or without reduction (ADDwnr) and osteophytes. In addition, the condylar morphology showed significant variation. Based on the severity of MRI findings at T1/CT1, 52.4% of patients were classified into stages 0–1, 9.5% into stage 2, and 38.1% into stage 3. Among the controls, the percentages were 72.2%, 16.7%, and 11.1%, respectively (p > 0.05). None of the patients’ or controls’ joints were classified into substages 2A, 2C, 3C, or 4. At T1/CT1, the total number of patients’ self-perceived symptoms at stages 1 and 3 varied from 1 to 21, and at stage 2 from 2 to 3. For controls, the number of symptoms ranged from 1 to 4 at all stages. Data on the MRI findings are presented in Table 5.

**Table 5.** Summary of MRI findings in patients’ and controls’ jaw joints before treatment (at T1/CT1), classified using the substages 1A–3B by Hegab et al. [30]. None of the joints were classified into substages 2A, 2C, 3C, or 4. Normal findings in **bold**. Distribution of patients’ and controls’ joints into the substages as follows:

	*Stage 0*	*Substage 1A*	*Substage 1B*	*Substage 2B*	*Substage 3A*	*Substage 3B*	*N*
Patient	1	8	29	4	0	10	52
Control	11	5	12	5	2	1	36
FINDING		Substage 1A	Substage 1B	Substage 2B	Substage 3A	Substage 3B	
DISC CONFIGURATION		JOINTS %	JOINTS %	JOINTS %	JOINTS %	JOINTS %	
**Normal (biconcave)**	Patient Control	**100** **100**	**72** **58**	**50** **60**	**0** **100**	**30** **0**	
Flattened	Patient Control	0 0	17 25	0 20	0 0	10 0	
Deformed	Patient Control	0 0	10 17	60 20	0 0	60 100	
DISC SIGNAL							
**Normal (evenly dark)**	Patient Control	**100** **100**	**31** **42**	**25** **0**	**0** **100**	**20** **100**	
Focal brightness in the disc	Patient Control	0 0	66 50	75 100	0 0	**50** **0**	
Not identified	Patient Control	0 0	3 8	0 0	0 0	30 0	
DISC POSITON							
**Normal**	Patient Control	**100** **100**	**100** **100**	**0** **0**	**0** **0**	**0** **0**	
Complete dislocation 10-8	Patient Control	0 0	0 0	100 100	0 100	100 100	
Posterior	Patient Control	0 0	0 0	0 0	0 0	0 0	
Missing	Patient Control	0 0	0 0	0 0	0 0	0 0	
Poor	Patient Control	0 0	0 0	0 0	0 0	0 0	
DISLOCATION							
**Normal (not dislocated)**	Patient Control	**100** **100**	**100** **100**	**0** **0**	**0** **0**	**0** **0**	
ADDwr^1^	Patient Control	0 0	0 0	100 100	0 0	0 0	
ADDwnr^2^	Patient Control	0 0	0 0	0 0	0 100	100 100	
Not identified	Patient Control	0 0	0 0	0 0	0 0	0 0	
Over	Patient Control	0 0	0 0	0 0	0 0	0 0	
Missing	Patient Control	0 0	0 0	0 0	0 0	0 0	
MUSCLE							
**Normal (no or single fat)**	Patient Control	**0** **0**	**83** **67**	**75** **80**	**0** **100**	**80** **100**	
Fat < Muscle	Patient Control	100 100	17 33	25 20	0 0	20 0	
Fat = Muscle	Patient Control	0 0	0 0	0 0	0 0	0 0	
Fat > Muscle	Patient Control	0 0	0 0	0 0	0 0	0 0	
CONDYLE POSITION							
**Normal**	Patient Control	**100** **100**	**90** **75**	**100** **80**	**0** **50**	**70** **0**	
Posterior	Patient Control	0 0	3 25	0 20	0 0	10 0	
Anterior	Patient Control	0 0	7 0	0 0	0 50	20 100	
CONDYLAR CORTEX							
**Normal (smooth, intact)**	Patient Control	**100** **100**	**34** **92**	**50** **80**	**0** **100**	**20** **100**	
Flattened	Patient Control	0 0	0 8	0 0	0 0	0 0	
Thicked	Patient Control	0 0	7 0	0 0	0 0	10 0	
Sklerotic	Patient Control	0 0	17 0	0 0	0 0	10 0	
Erosed	Patient Control	0 0	0 0	0 0	0 0	0 0	
Osteophyte	Patient Control	0	41 0	50 20	0 0	60 0	
FLUID							
**Normal, mild anterior**	Patient Control	**88** **100**	**100** **100**	**100** **100**	**0** **100**	**100** **100**	
moderate	Patient Control	12 0	0 0	0 0	0 0	0 0	
large	Patient Control	0 0	0 0	0 0	0 0	0 0	
CONDYLAR MORPHOLOGY							
**Normal (convex)**	Patient Control	**38** **40**	**7** **58**	**0** **80**	**0** **0**	**10** **0**	
Flattened	Patient Control	12 40	59 42	75 20	0 100	60 100	
Angeled	Patient Control	25 20	24 0	25 0	0 0	30 0	
Rounded	Patient Control	25 0	10 0	0 0	0 0	0 0	

^1^Anterior Disc Displacement with reduction.

^2^Anterior Disc Displacement without reduction.

### OQLQ

At T0/CT1, the range of patients’ OQLQ function scores varied from 5 to 20, and among controls, the scores ranged from 0 to 14. At the last time point (T5/CT3), patients’ OQLQ function scores ranged from 0 to 9 and those of controls from 0 to 19. From T0 to T5, one patient experienced a minor deterioration, her OQLQ function score increasing from 8 to 9. For 12 patients (92%), the OQLQ function score had diminished, with a mean improvement of 7.85 (median 7, range 2–17). At T5, the mean OQLQ function score had diminished to one-third (33%) of that at T0 (Table 2). From CT1 to CT3, the OQLQ function scores deteriorated in eleven (50%), did not change in five (23%), and improved in six controls (27%). For patients, the OQLQ sum score increased from a mean of 38.4 (median 34, range 7–82) at T1 to a mean of 46.8 (median 46, range 12–80) at T3, and decreased to a mean of 12.7 (median 5, range 1–40) at T5. (Additional data provided in references 30 and 31). For controls, the respective numbers were 18.2 (median 13, range 1–59) at CT1, 21.2 (median 11, range 0–70) at CT2, and 21.1 (median 17, range 0–65) at CT3.

### GSI

**Figure 1.**
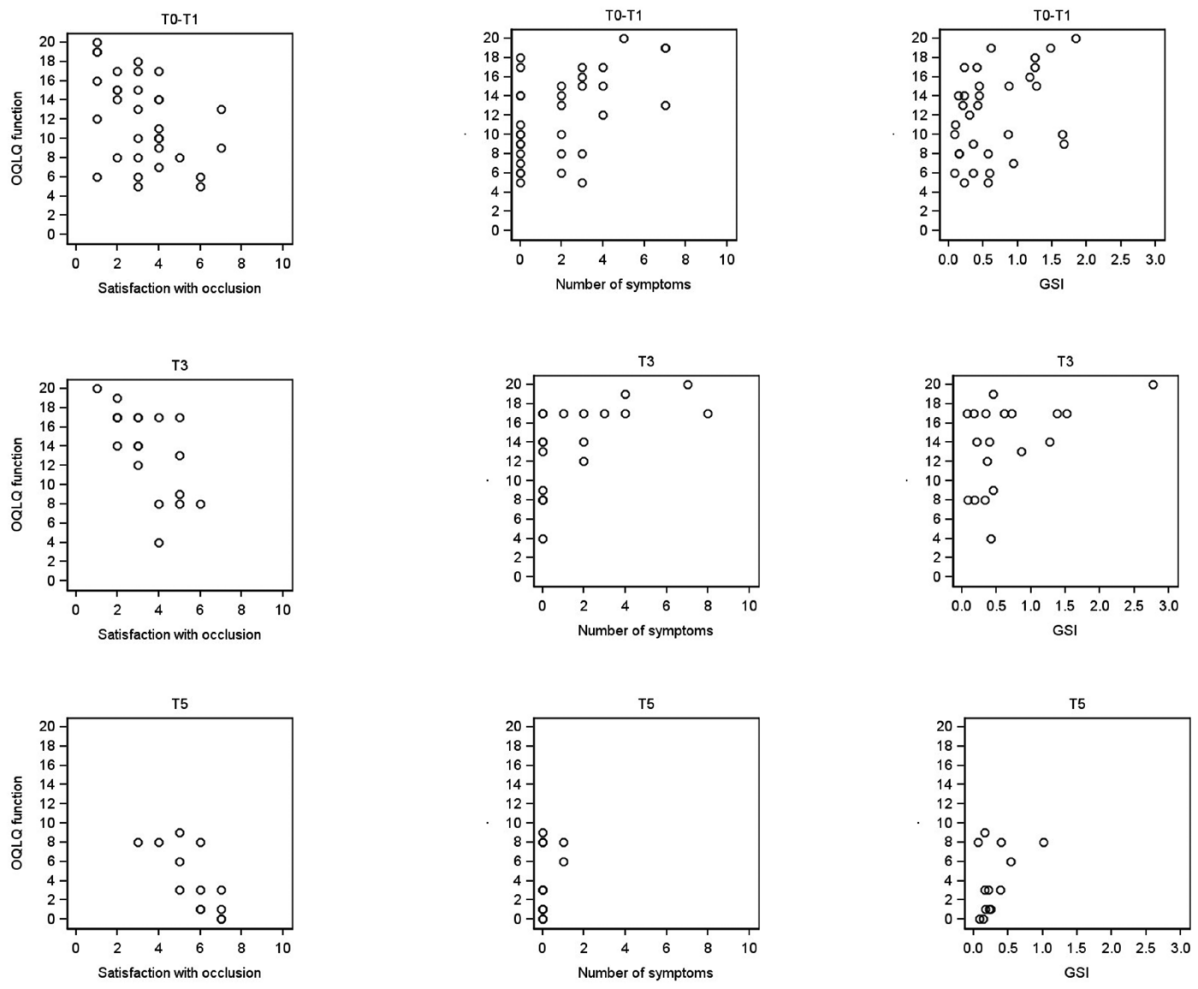
Correlations between patients’ OQLQ function scores, satisfaction with occlusal function, number of self-reported symptoms, and GSI before treatment (at T0–T1, n = 36), after presurgical orthodontics (at T3, n = 19), and one year after jaw surgery (at T5, n = 13).

Patients’ Global Severity Index (GSI), reflecting the mean score of the SCL-90, varied as the study progressed (Table 2). At T0, the GSI of 13 patients (36%) exceeded the national mean GSI value of adults (GSI 0.60, SD 0.44 [26]). At T3, the GSI of 8 patients (42%), and at T5, of one patient (8%) exceeded the mean value. (Additional data provided in references 30 and 31). The number of reported TMD symptoms positively correlated with the GSI at all time points, but at the initial stage T1, the correlation was not statistically significant (at T1, Spearman’s correlation (rho) 0.236, p = 0.166; at T3, rho 0.604, p = 0.006, and at T5, rho 0.628, p = 0.022). Correlations of OQLQ function and OQLQ sum score with GSI were significant at baseline (rho 0.377, p = 0.033 and rho 0.607, p < 0.001, respectively), while at T3, the correlation was significant between OQLQ sum score and GSI only (rho 0.661, p = 0.003). At baseline and T3, both OQLQ function and OQLQ sum score had significant negative correlations with the satisfaction with occlusal function (at T1, OQLQ function: rho −0.400, p = 0.023 and at T3, rho −0.680, p = 0.001; at T1, OQLQ sum score: rho −0.375, p = 0.035, and at T3, rho −0.811, p < 0.001), and positive correlations with the number of symptoms (at T1, OQLQ function: rho 0.476, p = 0.006, and at T3, rho 0.687, p = 0.001; at T1, OQLQ sum score: rho 0.429, p = 0.014, and at T3, rho 0.804, p < 0.001). None of the correlations between the classified MRI findings and satisfaction with occlusal function, OQLQ function or sum scores, number of symptoms or GSI were statistically significant (rho values ranging from −0.172 to 0.269, all p-values > 0.05) (Fig. 1). A case-by-case summary of the results is shown in Table 6.

**Table 6.** A summary of the combined data from the MRIs, change in total number of reported symptoms, OQLQ scores at T1 and T5, and change in satisfaction with occlusal function from T1 to T5 separately for each patient and control. GSI scores at T1 and T5 for patients only. Severity of MRI findings classified according to the method by Hegab et al. [30]. Distribution to the stages was as follows: Stage 0–1: 6 patients and 12 controls, Stage 2: 2 controls, and Stage 3: 7 patients and 2 controls. Satisfaction with own occlusal function assessed using a scale from 1 to 7, values 1–3 indicating dissatisfaction and values 4–7 satisfaction.

	Severity of MRI findings at T1	Change in number of symptoms at T5	GSI at T1	GSI at T5	GSI T1–T5	OQLQ at T1	OQLQ at T5	OQLQ T1–T5	Satisfaction with occlusal function at T5
PATIENTS	STAGES 0–1								
		−2	0.42	0.40	0.02	40	26	14	became satisfied
		−2	0.09	0.18	−0.09	14	5	9	remained satisfied
		−1	0.36	0.25	0.11	18	2	16	remained satisfied
		0	0.15	0.16	−0.01	34	20	14	remained satisfied
		0	0.09	0.07	0.02	15	13	2	remained satisfied
		−2	1.27	1.02	0.25	62	31	31	remained dissatisfied
	STAGE 3								
		−16	0.23	0.09	0.14	34	10	24	became satisfied
		+2	1.25	0.54	0.71	67	40	27	became satisfied
		−2	0.10	0.14	−0.04	36	1	35	remained satisfied
		0	1.25	0.23	1.02	63	5	58	remained satisfied
		0	1.67	0.39	1.28	45	5	40	remained satisfied
		0	0.44	0.22	0.22	27	3	24	remained satisfied
		0	0.60	0.16	0.44	8	4	4	remained satisfied
CONTROLS	STAGES 0–1								
		−4				29	31	−2	became satisfied
		−1				20	35	−15	remained satisfied
		−1				1	1	0	remained satisfied
		0				30	21	9	remained satisfied
		0				18	6	12	remained satisfied
		0				17	14	3	remained satisfied
		0				13	6	7	remained satisfied
		0				9	9	0	remained satisfied
		0				6	0	6	remained satisfied
		+1				7	15	−8	remained satisfied
		+4				59	65	−6	remained satisfied
		+8				12	21	−9	became dissatisfied
	STAGE 2								
		−1				9	16	−7	remained satisfied
		0				−	−		remained satisfied
	STAGE 3								
		−2				22	12	10	became satisfied
		−3				52	59	−7	remained dissatisfied

## Discussion

In TMD, occlusion or pathophysiologic changes in TMJ or its surrounding structures are not the only decisive factors [31]. The issue at stake is the multifactorial etiology, including, e.g. female gender, age, pain, physical trauma, facial symptoms, occlusion- and sleep-related factors, and psychosocial factors [32]. In TMD patients, pain intensity and pain disability have been found to associate with symptoms of depression and anxiety [33]. Likewise, psychological factors are associated with TMD-related pain prognosis [34].

As bruxism and other parafunctional factors, so, too, anxiety and depression independently increase the risk of TMD symptoms in the general adult population [35]. Furthermore, the relationship between TMD and dentofacial deformities is still controversial. In a recent study by Bonotto et al. [36], preoperative orthognathic patients’ TMD symptoms were evaluated in conjunction with, e.g. psychosocial factors and sleep quality. In their study, 54% of preoperative orthognathic patients had muscular TMD, while 44% had arthralgia. Patients were clustered into two groups, ‘vulnerable’ and ‘adaptive’, regarding painful TMD. Patients in the vulnerable group had significantly higher frequencies of muscular TMD, bruxism while awake, anxiety symptoms, and poorer sleep quality. Of these, somatic symptoms were the second- and anxiety the third-most significant predictor for painful TMD, suggesting that psychosocial variables play an essential role in this patient group. Also, the current results suggest that associations between GSI scores and TMD symptoms reflect an interplay between somatic wellbeing and psychological factors.

### MRI

The primary aims of this cross-sectional study were to analyze changes in patients’ self-reported TMD symptoms and investigate the associations between the TMJ-MRI findings, TMD symptoms, satisfaction with occlusal function, psychological distress, and impact on OQoL. The results were compared to those collected from voluntary control groups at respective time points (i.e. at the study beginning and twice later at two-year intervals). The TMJ-MRI findings were analyzed using the recently published system by Hegab et al. [30]. So far, it has not been used in other publications; thus, direct comparisons of the results are not yet possible. The system comprises a detailed view of degenerative changes in discs and condyles, disc displacement, joint effusion, and the state of the lateral pterygoid muscle. The findings are first classified into one of three groups based on disc displacement, and further, into one of five stages based on severity [30]. At T1, about one in two patients and three out of four controls were classified as having no disc displacement. However, they had some pathologic changes in the lateral pterygoid muscle, and possibly some joint fluid (1A) or degenerative changes in disc and/or condyle (1B). A total of 38% of patients and about 11% of controls had anterior disc displacement without reduction (ADDwnr), which is higher than the finding by Gaggl et al. [37] in preoperative patients. Instead, the share of ADDwnr in controls’ joints was less than half of that reported in asymptomatic controls by Eriksen et al. [38], but twice the number reported by Tallents et al. [39]. In the current study, ADDwr and ADDwnr were the primary and most significant findings, followed by disc deformation and advanced bone destruction. However, the whole is more important than individual findings. The Hegab system provides clinicians information on treatment modalities and prognosis at different stages of TMD [30].

Müller et al. [40] have reported about 60% agreement between the diagnoses of joints in orthodontic examinations and MRI studies. It has been suggested that MRI scans of the TMJs should be included in orthodontic-surgical patients’ examination protocol [41]. On the other hand, asymptomatic respondents, too, had findings in the MRIs. In contrast to the results by Li and Chang [42], but in line with those by Eriksen et al. [38], we did not find statistically significant associations between subjective symptoms and MRI findings. Nor was there any statistically significant correlation between the classified findings and satisfaction, GSI, OQoL, or the number of symptoms.

### Satisfaction with occlusal function

In general, patients who have received orthognathic treatment have been satisfied with the treatment. Recently, satisfaction rates of over 85% were reported by Zamboni et al. [43]. However, after preoperative orthodontics, our results show a statistically significant difference between patients’ and controls’ satisfaction with occlusal function. This is understandable: given that the primary goal of preoperative orthodontics is to reverse dental adaptations, some inconvenience and a momentary increase in symptoms can be expected. One year after surgery, after patients had sufficient time to recover and adjust to the new situation, there was no significant difference between the groups.

### Symptoms

Before surgery, patients reported the most TMD symptoms in the morning, and various symptoms affected two out of three patients. This finding aligns with the prevalence reported earlier among untreated adults with jaw discrepancies [13, 44]. As shown in the follow-up study by [28], the placement of fixed appliances further increased the OQLQ function scores, indicating more problems in biting and chewing. However, one year postoperatively, less than one in six patients in the current study reported TMD symptoms. Al-Riyami et al. [45] have also found similar trends in self-reported symptoms, although the authors emphasize the problem of statistical heterogeneity in the articles included in their review. The most frequently reported pretreatment symptoms were pain in the head and/or neck region, jaw tiredness and stiffness, difficulties chewing, and pain in TMJs. These findings, too, support those of Al-Riyami et al. [45], although the time points of data collection vary.

### OQLQ

One year postoperatively, patients’ OQLQ scores had decreased and were even lower than those of controls. Interestingly, based on current results, OQLQ sum score and psychological distress were also correlated. This may, however, be connected to several factors, such as pain and other bodily factors, satisfaction with dental appearance, or bullying. Thus, more studies are needed to clarify these interactions [46, 47].

This study differs from previous studies in following changes in TMD symptoms at different stages of orthodontic-surgical treatment and analyzing their association with changes in patients’ psychological distress and quality of life. The semi-structured diary was created for the longitudinal study [21]. Because the diary was filled in four times in one day, it provided detailed information about changes in TMD symptoms.

However, there were several limitations. Because of the small sample size, it is possible that some of the current results have not reached statistical significance. Therefore, the results shall be interpreted with caution. Increasing the number of participants would have required further lengthening to the recruitment time. At the beginning of the study, 3T MRI equipments were unfortunately not common and their use for research purposes was restricted.

Secondly, the focus of the current study was on females only. As shown by Olkun et al. [48], more females than males seek orthodontic-surgical treatment. In addition, females suffer from TMD more often than males, with a female-to-male ratio of 3.3:1 [49]. As a consequence, the current results cannot be directly generalized to male patients.

In this study, the control group comprised university students. This might have led e.g. to socioeconomic bias. However, unlike in many other countries, education in Finland is free of charge at all levels, from basic school to university studies. As a consequence, university students represent variable socioeconomic and demographic backgrounds.

From the methodological point of view, the recently published classification system by Hegab et al. [30] lacks wide validation. However, no single assessment method has been used as the gold standard in analyzing the TMJ-MRIs. To assist in possible comparisons, the current findings are provided also traditionally, at the TMJ level.

Nevertheless, orthodontic-surgical treatment is elective. Before prospective patients make the decision to start or reject treatment, it is clinicians’ responsibility to inform them about its possible pros and cons. This study adds to knowledge of the prevalence and variability of TMD symptoms during the treatment and the impact of symptoms on patients’ psychosocial well-being.

## Data Availability

Data supporting the study findings are available from the corresponding author upon reasonable request.
